# Plants from disturbed savannah vegetation and their usage by Bakongo tribes in Uíge, Northern Angola

**DOI:** 10.1186/s13002-016-0116-9

**Published:** 2016-09-20

**Authors:** Anne Göhre, Álvaro Bruno Toto-Nienguesse, Macaia Futuro, Christoph Neinhuis, Thea Lautenschläger

**Affiliations:** 1 Institut für Botanik, Technische Universität Dresden, D-01062 Dresden, Germany; 2 Universidade Kimpa Vita, Province of Uíge, Rua Henrique Freitas No. 1, Bairro Popular, Uíge, Angola

**Keywords:** Ethnobotany, Disturbance vegetation, Angola, Bakongo people, Traditional knowledge

## Abstract

**Background:**

This study represents the first in-depth ethnobotanical study in the province of Uíge in northern Angola and documents the traditional knowledge of the Bakongo people living in the area. Due to deforestation and frequent fires, degraded savannahs dominate the landscape in the study region. Here we provide a list of useful plants from these savannahs including quantitative data about cultural importance of the respective species, aiming on the one hand to conserve the local knowledge and on the other hand to create a reliable basis for research projects in the region.

**Methods:**

Field work was conducted in April and May 2014 in 5 municipalities of Uíge province. The study is based on 32 semi-structured and free-listing interviews, group discussions of varying scope and 14 field trips, involving a total of 82 informants. Throughout the course of the study herbarium specimens of the useful species were collected for later identification. Cultural importance index was applied to analyse the data sets recorded and to determine the best-known useful species in the region. All data sets were compared to the literature available for the region.

**Results:**

The study documents a total of 498 citations for the use of 122 plants from 48 families, 34.0 % of which were unknown according to the literature used for comparison. The high amount (71 %) of medical use-reports indicates that plants still play a crucial role in rural health care. We identified 14 plant species of special interest for pharmacological analysis. Species of highest cultural importance are *Annona senegalensis* Pers. and *Sarcocephalus latifolius* (Sm.) E.A., both of which are frequently found in disturbed savannahs.

**Conclusions:**

The study points out the importance of savannahs even if degraded in terms of useful plants and provides a valuable addition to current knowledge of plant use in Northern Angola. This is not only essential for further studies, i.e. regarding pharmaceutical agents, but also for the design of a planned botanical garden of the University Kimpa Vita in Uíge, which aims at communicating the findings to the local people.

## Background

Due to its botanical and cultural diversity, covering vegetation zones from tropical cloud forests in the north to the Namib Desert in the south [1] and many ethnic groups [2], Angola is a promising target for ethnobotanical research.

Previous studies already aimed at documenting ethnobotanical knowledge in Angola and preserving it for future generations. Leyens and Lobin [3] present portraits of Miombo and Mopane plant species and their uses, while Costa and Pedro [4] focus on medical plants and summarize data collected from across the country. Latham [5, 6], Kembelo [7] and Makumbelo [8] offer comparable information for the Bas-Congo-Province in the Democratic Republic of Congo, adjacent to the study area. However, systematic and comprehensive ethnobotanical analyses regarding the cultural and botanical diversity of the country are still scarce. The above-mentioned publications primarily document and propagate traditional knowledge, e.g. to improve health care for the local population, but do not include quantitative data and in some cases don’t give specific information about the ethnic groups using the plants. A recent study of Urso et al. [9] offers first quantitative data for the province of Namibe in the south-west of the country. Their survey emphasizes the importance of plants to both cultural identity and livelihood strategies in the area and identifies a high amount of species that were until then not known for their ethnobotanical importance. No comparable data are available for the north of the country. Due to 40 years of Civil War and War of Independence, a lot of research needs to be done in this field.

It was the aim of this study to document traditional knowledge about useful plants in the Province of Uíge in Northern Angola. The examined municipalities are widely dominated by savannah vegetation, severely degraded by frequent fires [1, 10]. Several ethnobotanical studies worldwide have stated that those disturbed areas are far more important to the local people than would be expected by means of species richness [11, 12]. Disturbed habitats are often found in the vicinity of human settlements, facilitating accessibility [12, 13], and contain a high percentage of weeds that are assumed to be of high medical value [14, 15]. The goal of this first in-depth ethnobotanical assessment of the region was to provide a list of the useful plants from savannahs also including quantitative data about cultural importance of species. By this means, the study aims to conserve local knowledge for future generations, to make it accessible for projects such as a planned botanical garden and to interpret it in the context of anthropogenic disturbance. Apart from that, our documentation intends to provide a reliable basis for future ethnobotanical and ethnopharmacological research in the region and to identify promising targets for such projects.

### Study area

**Fig. 1:**
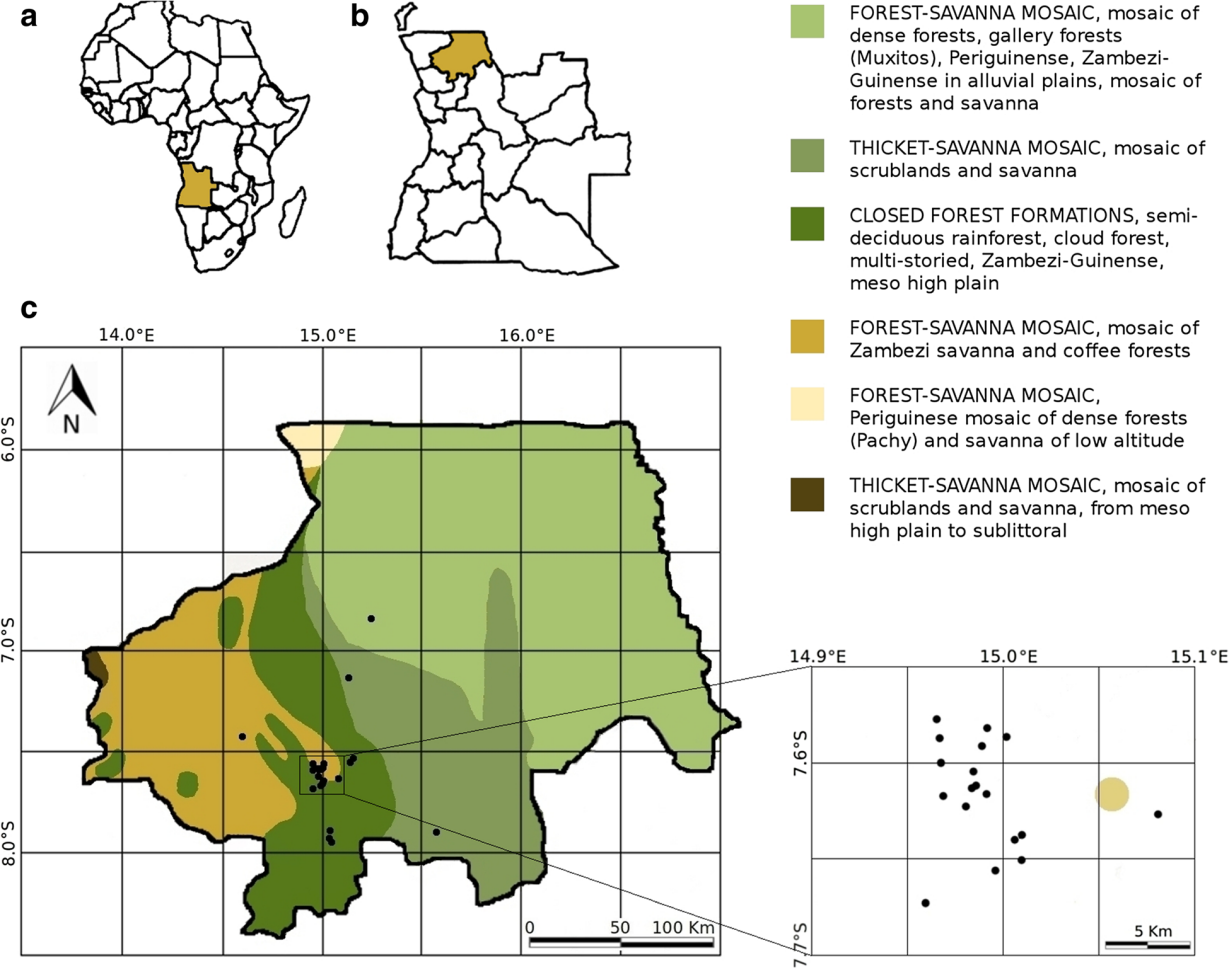
Map of study area. **a** Location of Angola in Africa **b** Location of Uíge in Angola **c** Vegetation zones in the province of Uíge according to [19]. Graphic: A. Kempe

Field work was carried out in the Province of Uíge in the north of Angola (Fig. 1). The study area extends in the range of S6°55'24.91" to S07°57'03.7'' and E14°36'14.7' to E15°30'27.02", mainly in the municipality of Uíge. Other municipalities covered by the study are Negaje, Quitexe, Mucaba and Ambuíla.

#### Climate and vegetation

Uíge Province is characterised by tropical wet and dry or savannah climate, classified by Köppen-Geiger as Aw [16, 17]. Despite four months of dry season extending from June to September [18], the surroundings of Uíge City were originally covered by closed forest formations and forest savannah mosaic [19]. The development of forests is favoured by a local climate in the neighbouring mountain chains of Serra do Uíge, Serra do Pingano and Serra Quibinda, where humid air masses condense during dry season [10, 20].

The forests in Uíge are under huge anthropic pressure: Timber exploitation, agriculture (mainly subsistence farming and slash-and-burn agriculture), urbanization and fragmentation of the habitat due to road construction lead to the degradation and loss of this habitat. The area of degraded savannahs increases, resulting in a mosaic of remaining cloud forests, secondary coffee forests and savannahs [1, 10, 19].

**Fig. 2:**
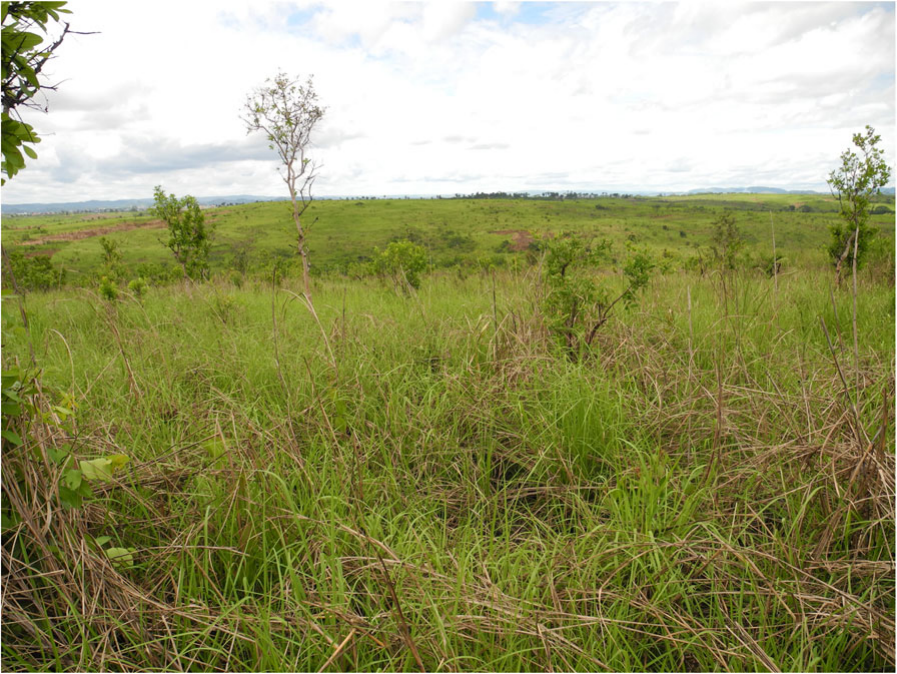
Degraded savannah landscape close to the city of Uíge. Study site close to the experimental farm of Kimpa Vita University

As illustrated in Fig. 1, most of the data sampling points are located in areas of potentially closed formations of semi-deciduous rainforest or evergreen cloud forests or in areas of secondary coffee forests and Zambesi savannahs. The actual vegetation is characterized by degraded savannahs, as seen in Fig. 2, which are widely dominated by the grass genus *Hyparrhenia*. Pyrophytic, rhizomatic herbs such as *Pteridium centrali-africanum* and *Aframomum alboviolaceum* and small, fire resistant shrubs such as *Psorospermum febrifugum*, *Bridelia ferruginea* and *Hymenocardia acida* are commonly found. The vegetation as well as interviews and personal observations lead to the conclusion that these savannah habitats are burnt on a regular basis. Different reasons for those large scale savannah fires were mentioned during interviews: Fires are meant to keep roads clear, to improve accessibility of grassland (e.g. to collect fruits of *Aframomum alboviolaceum*), to reduce agricultural pests, to create pasture land, to locate enemies earlier (during civil war) or are started without reasons or as a game.

Especially in interviews it was not always possible to differentiate between savannahs and other disturbed habitats in the vicinity of human settlements, such as village outskirts, fields, forest edges and recently burnt savannahs or forest patches. Also, field excursions sometimes included transition zones to those habitats, where vegetation is influenced by forest species, introduced or cultivated plants and/or ruderal species.

#### Population

The province of Uíge covers an area of 58.698 km^2^ and counts about 1.4 million inhabitants [21]. The majority of the local population belongs to the Bakongo ethnic group. This Bantu group lives in an area that covers parts of what is now Angola, Democratic Republic of the Congo, Republic of the Congo and Gabon. They are united by the Bantu-language Kikongo, although the wide distribution, different colonial powers, transregional trade and migration have lead to a great diversity in spoken and written forms of this language [22]. In Angola most persons additionally speak the official language Portuguese.

In the rural areas involved in the study, agriculture is the main source of food and income. Small subsistence family farms are prevailing [1], information on field size ranges between 1.4 ha and 2.25 ha [23, 24]. Most smallholders practise shifting cultivation. As mechanization is uncommon, the land is mostly prepared manually and fire is often used to remove biomass (slash-and-burn agriculture) [1].

Western medical health care is scarce, especially in rural areas [25]. The under-five mortality rate in Angola is the highest in the world, with 157 deaths per 1000 live births [26]. Curent economic problems due to the low oil price might aggravate the situation. It is assumed that importance of traditional healers and herbal medicines especially for rural people is high and might even increase in the coming years [9, 27].

## Methods

Field work was conducted between April 6th and May 24th, 2014. The study is part of an academic cooperation between Technische Universität Dresden and Universidade Kimpa Vita. Permits for research, collection and export of voucher specimens were obtained from Instituto Nacional da Biodiversidade e Áreas de Conservação of the Ministério do Ambiente da República de Angola and from Direcção Provincial da Agricultura, Desenvolvimento Rural e Pescas of the Governo Provincial do Uíge.

Prior to interviews and field excursions all participants in the study were informed about the project and their rights and local authorities were asked for permission to work within the community and the surroundings (prior informed consent). Communication was mainly conducted in Portuguese. If necessary, Angolan co-workers or village residents translated into Kikongo. The methods used for ethnobotanical data collection included semi-structured interviewing, free-listing, group discussions regarding plant identification and field excursions. Data sets collected included the vernacular name of the plant in Kikongo and/or Portuguese, usage and the plant parts used as well as preparation and administration techniques. Data sets collected during interviews were only included in the analysis if they could be assigned to plants identified during field trips.

During field excursions and subsequent to interviews, herbarium specimens were collected for later identification. All voucher specimens are deposited in the Herbarium Dresdense (DD) of the Institute of Botany, Technische Universität Dresden, Germany. As soon as suitable conditions are established, type species and duplicates of the collection will be deposited at Universidade Kimpa Vita, Uíge, Angola. Identification of plant species was carried out at the Institute for Botany of TU Dresden. If it was not possible to collect herbarium specimens, photographs of the plants were used. The following floristic works were consulted for identification: Conspectus Florae Angolensis [28–30], Plantas de Angola [31], Flora of Tropical West Africa [32–36], Flore Analytique du Bénin [37] and Flora Zambesiaca [38]. Additional information was retrieved from Kew Herbarium Catalogue [39], Herbario LISC [40] and Herbarium Dresdense.

Because of time constraints, not all plants were found with flowers and fruits, impeding the determination to species level. The associated use-reports were only included in the results if the specimen was at least determined to family level.

In total, 41 informant groups were involved in the study. These groups were made up of 82 individuals between the ages of 23 and 80 years, with 56 % of informants being women. We conducted 32 interviews and 14 field trips with these informant groups. Group size varied between one and nine persons in interviews, with a mean value of 2.3. In field excursions, group size varied between one and four participants, with an average value of 1.8. Whether field excursions and interviews were carried out with single persons or groups was mainly influenced by decisions of local authorities.

To establish contact with potential informants, local authorities were asked to suggest persons with different backgrounds that might participate in the study. The great majority (74 %) of informants were farmers who were known within the community for having knowledge about the use of plants. 10 % of participants belonged to the group of local authorities, who were sometimes also involved in farming activities. A further 7 % of participants were traditional healers or midwives, 6 % workers or employees (non-farming activity), while teachers within the villages represented 3 %. To quantify ethnobotanical data, the information regarding useful plants was organised in use-reports. Use-reports follow the basic structure of ‘informant [group] i mentions the use of species s in the use-category u’ [41, 42]. Use-categories were adopted from the data received during the interviews and included: nutrition/food, medicine, tea, ornamental, ritual/magic, forage, fibres, handicraft/building material and fuel. Use categories mentioned less than 4 times (e.g. fish poison, candles) were summarised in the residual category “others”.

Use reports were analysed calculating the number of use-reports (NUR) and the cultural importance index (CI) (Formula 1). The NUR is one of the most commonly used tools to measure the cultural importance of plants [41]. It is calculated by firstly summing the number of informants who mention each use-category for the species and secondly summing the values of each use-category [41]. The CI was introduced by Tardío and Pardo-de-Santayana [41]. It is comparable to the use value, which is widely applied in ethnobotanical research [13, 43]. Unlike NUR, it is not influenced by the total number of informants and therefore allows the comparison of data between different studies. As informants were mostly interviewed in groups and use reports were only recorded once per group, we used the number of the interviewed groups substitutional for the total number of informants in our calculation.

To give more accurate information about the use of plants, large use-categories were divided into sub-categories or usages. In the medical use-category, subcategories were determined by the illness or symptom treated with the plant. Subcategories were documented without further grouping of the cited usages or illnesses. For food plants, subcategories were defined by the plant part and the preparation method used. Subcategories mentioned during the interviews included: Raw fruits (eaten fresh or dry), cooked fruits, cooked leaves (eaten as vegetable), cooked tubers and raw stem.

If one informant mentioned the use of a species in two subcategories belonging to the same use-category, those use-reports were counted as one data-set in the use-category for calculation of NUR and CI. E.g., if one informant mentioned the use of a species against abdominal pain and diarrhoea, those use-reports counted as one data-set in the category “medicine”. This was necessary because many use-reports, especially for medical uses, were closely related, which could result in an overestimation of cultural importance. Because of the introduction of subcategories, NUR per species does not always equal the sum of all use-reports listed in the sub-categories.

$$ NU{R}_s\kern0.5em =\kern0.5em {\displaystyle \sum_{u={u}_1}^{{}^uNC}{\displaystyle \sum_{i={i}_1}^{{}^iNI}U{R}_{ui}}} $$

$$ C{I}_s\kern0.5em =\kern0.5em {\displaystyle \sum_{u={u}_1}^{{}^uNC}{\displaystyle \sum_{i={i}_1}^{{}^iNI}\frac{U{R}_{ui}}{NI}}} $$

Formula 1: (A) Calculation of number of use-reports and (B) calculation of cultural importance index according to [41]. *NUR* = Number of use-reports, *CI* = cultural importance index, *s* = species, *u* = use-category, *i* = informant, *NI* = total number of informants, *NC* = number of use categories, *UR* = use-report.

To evaluate the increase of knowledge through the study, all data sets were compared to available literature. For use-reports referring to medical treatments Neuwinger [44], for all other applications Latham [45] and Latham and Konda ku Mbuta [5] were used for comparison. To estimate the potential for further medical studies, a PubMed inquiry (http://www.ncbi.nlm.nih.gov/pubmed) was carried out in June 2015 for each species to determine if medical studies have already been carried out.

## Results

The study documents a total of 498 different use-reports for 122 plants from 48 families. All use-reports are summarised and provided in Table 1. The most commonly used plant families are Fabaceae (13.1 %), Asteraceae (13.1 %), Euphorbiaceae (6.6 %), Lamiaceae (6.6 %) and Malvaceae (5.7 %), all of which are widely distributed in Angola [31].

**Table 1:** Overview of the documented useful plant species and their uses sorted by plant families

UC^a^	Subcategory, explanation of usage	PP^b^	Preparation, Explanations	Administration	N^c^	CI^d^	L^e^	DB^f^
Acanthaceae								
*Acanthus montanus* (Nees) T.Anderson; +; 042677					1	0.02		M, C
M	Skin disease	L	Infusion	Dermal	1		o	
*Brillantaisia owariensis* P.Beauv.; Lemba-lemba; + 044075					4	0.10		M
M	Cardiac disease	L	Decoction (with sugar)	Oral	1		o	
M	Childhood disease	L		Bath	2		-	
M	Blood pressure (high or low)	L	Directly consumed	Oral	1		o	
M	Abdominal pain	L	Directly consumed	Oral	2		+	
M	Childhood disease (Gota)	L	Cold water extract	Oral	1		-	
M	Eye disease	L	Directly consumed	Oral	1		-	
Amaranthaceae								
*Dysphania ambrosioides* (L.) Mosyakin & Clemants; Santa Maria, Nkavua; * (*Chenopodium ambrosioides*), 042698					10	0.24		M, C
M	Abdominal pain	L		Enema	1		o	
M	General	L	Directly consumed	Oral	2		-	
M	Respiratory disease	L	Infusion	Oral	1		+	
M	Backache and rheumatic pain	L	With *Milletia versicolor, Ocimum gratissimum* and *Persea americana*	Steam Bath	1		+	
M	Fever	L	With *Cyperus* sp., *Xylopia aethiopica*, *Monodora myristica*, Kafuke (Asteraceae indet.),	Enema	1		+	
M	Gynaecological disorder	L	With *Chromolaena odorata* and *O. gratissimum*		1		+	
M	Malaria	L	With *X. aethiopica*, Kafuke (Asteraceae indet.), *M. myristica, O. gratissimum*		1		o	
M	Childhood disease (Growth disorders)	L	With *Ageratum conyzoides* and *O. gratissimum*	Dermal	1		o	
M	Diarrhoea	L	(A) Directly consumed (B) with *X. aethiopica*, Kafuke (Asteraceae indet.), *M. myristica, O. gratissimum*	Oral	2		o	
Anacardiaceae								
*Lannea cf. antiscorbutica* (Hiern) Engl.; Nkumbi; +; F10-1961, 043178					4	0.10		--
M	Toothache	B	(A) Decoction of crushed bark (B) with *Carica papaya*	(A) Applied to tooth	2		/	
M	Pulled muscles, fractures	B	Decoction of crushed bark	Applied externally and left to dry, forms cast	3		/	
Anisophyllaceae								
*Anisophyllea* cf. *quangensis* Engl. ex Henriq; Mfungua, Bilasoba; +; 043115					2	0.05		--
M	Skin disease	R	Balm is made after bark is removed	Dermal	1		/	
N	Fresh fruit	F	Directly consumed		1		+	
Annonaceae								
*Annona senegalensis* Pers.; Nlolo, Nlolo kambulu, Nlolopolo, Nloloa pequena, Mfuilu; +; 043115					16	0.39		M, C
M	Menstrual disorder	R	Decoction	Oral	1		+	
M	Haemorrhoids	R	Crushed root	Rectal	1		-	
M	Fertility	R, L, B	Infusion	Oral	4		+	
M	Hernia	R	Decoction	Oral, 2 cups daily	1		-	
M	Leg pain	R, L	Infusion	Oral	1		o	
M	Abdominal pain	R, L	Decoction	Oral or enema	2		+	
M	Worms	R, L	Infusion	Oral	2		+	
M	Respiratory disease		With *Aframomum alboviolaceum*		1		+	
N	Fresh fruit	F			1		+	
T		L			4		-	
*Monodora myristica* (Gaertn.) Dunal; Peve, Gipeve, Gipehe; +; 042679					8	0.20		M, C
M	Cough	S	Directly consumed	Oral	1		+	
M	Malaria	S	With *X. aethiopica*, Kafuke (Asteraceae indet.), *D. ambrosioides*, *O. gratissimum*		1		o	
M	Diarrhoea	S	with *X. aethiopica*, Kafuke (Asteraceae indet.), *D. ambrosioides*, *O. gratissimum*		1		o	
M	Abdominal pain	L, S	Sometimes on combination with other species	Enema	3		+	
M	Backache	S	With *Ochna* cf. *afzelii* subsp. *mechowiana., M. myristica* and Kupidi (*Piper* sp.)		1		o	
M	Fever	S	With *Cyperus* sp., *X. aethiopica*, *D. ambrosioides*, Kafuke (Asteraceae indet.),	Enema	1		+	
M	Worms	S	Infusion with other species, e.g. Kupidi (*Piper* sp.), *X. aethiopica* and Ndungu za nzó (*Aframomum* sp.)	Oral	1		+	
Apiaceae								
*Steganotaenia araliacea* Hochst.; Mumvumbimvumbi; +; 043212					1	0.02		M, C
M	Thrombosis	L	Decoction	Dermal	1		-	
M	Fertility	B, R	Decoction of 1,5 spoon of powder in 1 l of water	Oral, 2 cups daily	1		+	
Apocynaceae								
*Landolphia owariensis* P.Beauv.; Macongue, Makonge; +; 043969					6	0.15		M
N	Fresh fruit	F	Directly consumed		6		+	
Asteraceae								
*Acanthospermum* sp.; Makoloko; F09-1725					1	0.02		
M	Headache	P	Directly consumed	Chew	1		+	
*Ageratum conyzoides* (L.) L.; Kambwa katela, Mbokatela, Mbukata;+; 043150					5	0.12		M, C
M	Fever	P	Decoction	Bath	2		+	
M	Eye disease	L	Leaf sap from crushed leaves	Applied to eye	1		+	
M	Childhood disease (Growth disorders)	L	With *O. gratissimum* and *D. ambrosioides*	Dermal	1		o	
M	Abdominal pain				1		+	
*Baccharoides guineensis* (Benth.) H.Rob.*;* Matita, Matitita, + (as *Vernonia guineensis* Benth.); 043110					4	0.10		M, C
M	Worms	R	Cold water extract from crushed root tubers	Enema	1		o	
M	Childhood disease (Baço)	R	Directly consumed (peeled root tubers)	Oral	1		o	
M	Wounds	R	Directly consumed (peeled root tubers)	Oral	1		+	
M	Hernia	R	Directly consumed (peeled root tubers)	Oral	1		+	
M	Abdominal pain	R	Directly consumed (peeled root tubers)	Oral	1		o	
*Bidens pilosa* L.; Potajambua; +; 042705					1	0.02		M, C
T		L			1		+	
*Chromolaena odorata* (L.) R.M.King & H.Rob.; Mobutu, Kongo dia sika, Mululusaire; - (*); 042708					7	0.17		
M	Gynaecological disorder	L	With *O. gratissimum* and *D. ambrosioides*		1		o	
M	Abdominal pain	L	(A) Cold water extract of crushed leaves (B) crude crushed leaves	(A) Oral/enema (B) Compress (abdomen)	2		-	
M	Wounds	L	Crushed leaves	Dermal, applied to wound	3		+	
Ot	Soil fertility	P	Planted in *Manihot esculenta* field		1		+	
*Crassocephalum rubens* (Juss. ex Jacq.) S. Moore; Bungudia; +; 044078					8	0.20		M
N	Cooked leaves	L	Cooked and eaten as a vegetable		5		+	
Fo		L	For rabbits and pigs		3		-	
*Emilia coccinea* (Sims) G.Don; Malalulalu; +; 044086, 044087					3	0.07		M, C
M	Wounds	L	Leave sap from crushed leaves	Dermal, applied to wound	1		+	
Fo		L	For rabbits		1		+	
M	Eye disease	L	Leave sap from crushed leaves	Rubbed into eyes	1		+	
*Galinsoga quadriradiata* Ruiz & Pav.; Kabuata branca; (*); 043141					1	0.02		M
M	Hepatitis	L	Decoction	Oral	1		-	
*Gymnanthemum* cf. *glaberrimum* (Welw. ex O.Hoff.) H.Rob.; Nsalu; Kisalu; 044039					4	0.10		--
M	Anaemia	R	Decoction of crushed root	Oral	1		-	
M	Scabies	L	Ash	Dermal	1		+	
M	Backache	R	Cold water extract	Oral	1		-	
M	Worms	R	Cold water extract	Oral	1		-	
M	Abdominal pain	R, L	(A) Crushed root (B) Infusion	(A) Dermal (B) Oral	3		+	
*Helichrysum mechowianum* Klatt; + (var. *mechowianum*); 044088					1	0.02		--
Ot	Toilet paper	L			1		-	
--; Kafuke; 044034					7	0.17		
M	Abdominal pain	L	Sometimes with e.g. *X. aethiopica*, *M. myristica*	Enema	3			
M	Childhood disease (light stomach pain)	L		Enema	1			
M	Diarrhoea		With *D. ambrosioides*, *X. aethiopica*, *M. myristica, O. gratissimum*		1			
M	Fever	R	With *Cyperus* sp., *X. aethiopica*, *D. ambrosioides*, *M.myristica*	Enema	1			
M	Malaria				1			
M	Fertility	L	With *Urena lobata*	Enema	1			
M	Worms	L		Enema	1			
*Melanthera scandens* (Schumach. & Thonn.) Roberty; Kalahi, Kalau, Makaila; +; 042782, 042778					2	0.05		M, C
M	Wounds	L	Crushed leaves	Dermal, applied to wound	2		+	
*Pleiotaxis rugosa* O.Hoffm.; Ntelamakatexe, Kakatiana; 044020					3	0.07		--
M	Abdominal pain	L, R	Directly consumed or infusion	Oral, not during pregnancy	2		/	
M	Diarrhoea	L, R	Directly consumed (root) or infusion (leaves), masticate root		1		/	
*Polydora serratuloides* (DC.) H.Rob.;+ (listed as *Vernonia perrottetii* Sch. Bip. ex Walp.); 042793					1	0.02		--
O		P	Planted on graveyards		1		/	
*Tithonia diversifolia* (Hemsl.) A.Gray; Mululula; *; 044090					1	0.02		M, C
M	Abdominal pain	L	Crushed leaves	Compress (abdomen)	1		+	
*Vernonella subaphylla* (Baker) H.Rob. & Skvarla; Makutula; + (listed as *Vernonia subaphylla* Baker); 044024, 042795, 042797					5	0.12		--
T		L			3		/	
M	Skin disease (furuncle)	L	Infusion	Oral	2		/	
Bixaceae								
*Bixa* cf. *orellana* L.; Ndalamuenga; *; F03-131					3	0.07		M, C
M	Childhood disease (Baço)	B	Decoction	Oral	1		-	
M	Abdominal pain	R	Directly consumed or cold water extract	Oral	2		o	
M	Malaria	B	Decoction	Oral	1		-	
Ot	Cosmetics		Used for red colour		1		+	
Burseraceae								
*Canarium schweinfurthii* Engl.; Mbidi, Gimbidi; +; F09-1730, F09-1734					6	0.15		M, C
N	Cooked fruit	F	Left in warm water for 1 h or until pulp softens		1			
M	Respiratory disease	Re		Rubbed on breast, inhalation	1		+	
M	Toothache	L	Decoction with *Psidium guajava* and *Alchornea cordifolia*	Rinsed in mouth	1		-	
M	Abdominal pain	Re, R	(A) heat resin and mix with palm oil, *X. aethiopica* and *M. myristica* (B) clean (and crush) crude root	(A) Add to food (B) Oral or enema	2		+	
M	Nightmares	Re	Burnt	Inhalation	1		-	
Ot	Candles	Re	Used as wax substitute		1		o	
*Dacryodes edulis* (G.Don) H.J.Lam; Safueiro, +, F02-1181					2	0.05		M, C
N	Cooked fruit	F	Heated in water or in pan until pulp softens		2		+	
Cannaceae								
*Canna indica* L.; Chala (verde), Cholo; *; 042701					2	0.05		M, C
M	Thorax pain	L	Whole leaves	Compress at abdomen	1		-	
H		S	For rattles		1		+	
Caricaceae								
*Carica papaya* L.; Mamoeiro; *; F10-1978					4	0.10		M, C
M	Abdominal pain	L	Crushed leaves	Compress at abdomen	1		+	
M	Nausea	L	Crushed leaves	Compress at abdomen	2		o	
M	Toothache	R	Decoction, (sometimes with *Lannea antiscorbutica*)	Rinsed in mouth	3		+	
Caryophyllaceae								
*Drymaria cordata* (L.) Willd. ex Schult.; Lumpwua; +; 042678					1	0.02		M, C
M	Hepatitis	L, P	Crushed material	Dermal or enema	1		o	
Commelinaceae								
*Commelina diffusa* Burm.f.; Ndakalaka; +; 044029					1	0.02		M
M	Eye disease	L	Crush leaves with three salt crystals, add some water for the crystals to dissolve.	Applied to eye, three drops two times daily	1		o	
Costaceae								
*Costus afer* Ker Gawl.; Nsangalavula; +; F08-1918, F06-1709					7	0.17		M, C
M	Gota	St		Bath (also for children)	2		o	
M	Hepatitis	St	Directly consumed	Oral (eat like sugar cane)	1		-	
M	Measles				1		-	
M	Eye disease				2		+	
M	Yellow fever				2		-	
M	Strengthening	St	Directly consumed	Oral (eat like sugar cane)	1		-	
N	Raw stem	St	Directly consumed	Oral (eat like sugar cane)	1		o	
Crassulaceae								
*Kalanchoe crenata* (Andrews) Haw.; Luikiaikuai; +; 042667					2	0.05		M
R		P	Part of the ceremony to ask the ancestors for permission to enter the Grutas do Nzenzu		1		-	
M	Earache	L	Sap of crushed leaves	Applied to ear	1		+	
Cucurbitaceae								
*Lagenaria* sp.; Disenga; 043144					1	0.02		
H		F	Dried fruit is opened and used as brush		1			
*Luffa cylindrica* (L.) M.Roem.; Nzenga-nzenga; +; 042712					1	0.02		M, C
H		F	Dried fruit used as sponge		1		+	
*Momordica charantia* L.; Lumbuzam-buza, Nlumbuzu-buzua; +; 042663					4	0.10		M, C
M	Sore throat	P	Directly consumed	Wrapped around neck	1		-	
M	Fever	L	Decoction	Enema	2		+	
M	Constipation	L	Decoction	Enema, for children	1		+	
M	Typhoid fever	L	Decoction	Oral or enema	2		-	
Cyperaceae								
*Cyperus* sp.; Nsagonsago; F09-1717 to F09-1721					1	0.02		
M	Fever	R	With *M. myristica*, *X. aethiopica*, *D. ambrosioides*, Kafuke (Asteraceae indet.)	Enema	1			
Dennstaedtiaceae								
*Pteridium centrali-africanum* (Hieron.) Alston; Manguelele, Mizili, Manzelele; 043242					5	0.12		--
N	Cooked leaves	L	Young fronds are cooked as side dish (Mitekwa)		4		+	
M	Epilepsy	L, Rh	Decoction	Bath or enema	1		-	
Dioscoreaceae								
*Dioscorea dumetorum* (Kunth) Pax; +; 044023					1	0.02		M, C
N	Cooked root tubers	R			1		+	
Euphorbiaceae								
*Alchornea cordifolia* (Schumach. & Thonn.) Müll.Arg.; Luunze; +; 043214					5	0.12		M, C
M	Fever	L	Crushed leaves	Dermal	1		+	
M	Skin disease (furuncle)	F	Directly consumed	Oral, swallowed in whole	1		+	
M	Toothache	L	Decoction with *Psidium guajava* and *Canarium schweinfurthii*	Rinsed in mouth	3		+	
*Euphorbia cotinifolia* L.; - (*); F11-2000 to F11-2004					1	0.02		C
O		P			1		/	
*Euphorbia hirta* L.;- (*); 042704					1	0.02		M, C
M	Eye disease	Ms	Latex from sprout	Applied to eye	1		+	
*Euphorbia pulcherrima* Willd. ex Klotzsch;*; 042700					3	0.07		M, C
M	Childhood disease (blood loss)		With *Maesa* sp.		1		/	
M	Inflammation	Ms	Latex from sprout	Dermal	1		/	
O		P			1		/	
*Euphorbia thymifolia* L.; Mayene mankombo; - (*); 042692					2	0.05		M, C
M	Eye disease	Ms	Latex from sprout	Applied to eye	1		-	
M	Childhood disease (Diarrhoea)	P	Decoction	Oral, drunken by mother, cleans breast milk	1		-	
*Jatropha curcas* L.; Mpuluka; *; F10-1959					4	0.10		M, C
N	Cooked fruits	F	Cooked		1		-	
M	Toothache	L	Decoction	Rinsed in mouth	3		+	
*Neoboutonia melleri* (Müll.Arg.) Prain; Kiunze, Luunze, +; 043156					3	0.07		C
M	Diabetes	R	Decoction	Oral or enema	1		-	
M	Diarrhoea	R	Decoction	Oral or enema	1		-	
M	Abdominal pain	L	Crushed with salt	Oral	1		o	
*Ricinus communis* L.; Mamonoa; *;042668					3	0.07		M, C
M	Nausea	L	Cold water extract of crushed leaves	Oral	1		+	
M	Headache	L	crushed leaves	Applied to head, compress	3		+	
Hypericaceae								
*Harungana madagascariensis* Lam. ex Poir.; Ntunu; +; 043193					5	0.12		M, C
D	Yellow or orange dye	B	Cold water extract		3		+	
M	Yellow fever	B	Decoction with e.g. *Erythrina abyssinica*	Enema (causes strong, cleaning diarrhea)	2		+	
M	Hepatitis	B	Decoction with e.g. *Erythrina abyssinica*	Enema (causes strong, cleaning diarrhea)	1		+	
*Psorospermum febrifugum* Spach; Nlengula, Kilengula, Pau preto; +;042683					5	0.12		M, C
M	Leprosy	R	Crushed root	Dermal	1		+	
M	Fertility		Decoction	Oral	1		-	
M	Skin disease	B	Crushed bark	Dermal	2		+	
M	Childhood disease (Baço)				1		o	
Iridaceae								
*Eleutherine* cf. *bulbosa* (Mill.) Urb.; - (*); 043138					1	0.02		M, C
O		P			1		+	
Lamiaceae								
*Alvesia rosmarinifolia* Welw.; +; 042670					1	0.02		--
M	Anaemia	L	Crushed leaves	Dermal	1		o	
*Clerodendrum formicarum* Gürke; Lomba a mvula (pequena); +; 042662					4	0.10		C
M	Yellow urine	L	Infusion	Oral	1		/	
M	Childhood disease (Abdominal pain)	L, R	Infusion	Oral or enema	3		/	
*Hyptis suaveolens* (L.) Poit.; Kinsaquati; - (*); 043097					1	0.02		M, C
M	Fever	L	Infusion	Inhalation, steam bath	1		o	
indet.; 043148					1	0.02		
M	Eye disease		Liquid from crushed material	Applied to eye	1			
*Leonotis* sp.; Kakenginzongo; F08-1805					1	0.02		
M	General	L	Infusion	Oral	1			
*Ocimum gratissimum* L.; Mazudizudi, + (as var.); 042719					11	0.27		M, C
M	Childhood disease (Growth disorders)	L	With *Ageratum conyzoides* and *D. ambrosioides*		1		o	
M	Fever, Malaria	L	Infusion of crushed leaves	Oral or steam bath	5		+	
M	Backache, Rheumatic pain	L	With *Milletia versicolor, D. ambrosioides* and *Persea americana*	Steam bath	1		+	
M	Gynaecological disorder	L	With *C.odorata* and *D.ambrosioides*		1		+	
M	Diarrhoea		With *D. ambrosioides*, Kafuke (Asteraceae indet.), *M. myristica, X. aethiopica*		1		+	
M	Respiratory disease	L	Infusion of crushed leaves	Oral or steam bath	4		+	
*Vitex madiensis* subsp. *madiensis*; Mafilu, Mfilumfilu; +; 042773					10	0.24		M
M	Backache	L	Infusion	Oral	3		-	
M	Childhood disease (Gota)	L	Infusion, sometimes with *Gardenia ternifolia* and *Smilax anceps*		2		o	
M	Potency, Strengthening (for women and men)		Infusion	Oral	1		+	
T		L			1		-	
N	Fresh fruits	F			2		+	
Fo			Pasture for cattle		1		-	
Leguminosae								
*Albizia* sp.; Mulu, Muanse; 043102					2	0.05		
M	Headache	R	Liquid from crushed root	Applied into nose	1			
M	Diarrhoea	B	Cold water extract from crushed bark	Enema	1			
*Bauhinia variegata* L.; - (*); F11-2064					1	0.02		M, C
O					1		/	
*Cajanus cajan* (L.) Millsp.; Wandu; *; 043216					2	0.05		M, C
M	Childhood disease (Gota)	L	Liquid from crushed leaves	Applied into nose and eyes	1		o	
N	Cooked fruits	F			1			
*Calopogonium mucunoides* Desv.; *; 042672					1	0.02		C
Ot	Soil fertility	P	Planted in crop area		1		/	
*Canavalia* cf. *gladiata* (Jacq.) DC.; Nzimamanu; - (*; origin undeterminated); F01-1142 to F01-1144					4	0.10		M, C
R	Protection against mischief	F, P	(A) Fruit hung around wrist of big children prevents defamation (B) Plant at the edge of field protects it against influence of bad neighbours		3		+	
M	Childhood disease	F	Fruit hung around wrist of crying babies		1		-	
*Desmodium velutinum* (Willd.) DC; Malamalama; +; 044091, 044092					1	0.02		--
M	Headache	L	Crushed leaves	Dermal, applied to head	1		-	
*Eriosema* glomeratum (Guill. & Perr.) Hook.f.; Zila wando; +; 043170					1	0.02		C
M	Diarrhoea	L	Directly consumed	Oral	1		o	
*Erythrina abyssinica* DC.; Mulungulungu, Mungomangoma, Nlungwa kwma; +; 042666, 043211					10	0.24		M, C
M	Hepatitis	B	(A) Infusion (B) decoction with i.a. *Harungana madagascariensis* (C) Cold water extract from crushed bark	(A, C) Oral (B) Enema, causes cleaning diarrhoea	7		o	
M	Yellow fever	B	(A) Infusion (B) decoction with i.a. *Harungana madagascariensis*	(A) Oral (B) Enema, causes cleaning diarrhoea	2		o	
M	Typhoid fever	B	Infusion	Oral	2		-	
M	Fertility	B	Decoction with sugar	Oral	1		+	
M	Backache				1		o	
indet.; Monguenia; 043096					3	0.07		
M	Backache	B	Crushed bark	Enema	1			
M	Diarrhoea	R	Cold water extract	Enema	1			
M	Abdominal pain	R	Cold water extract	Enema	1			
indet.; Musuemba; 043103					2	0.05		
D	Dye				1			
Ot	Fish poison				1			
indet.; Musoshi; 043133					1	0.02		
M	Burns	L	Balm from ash mixed with palm oil	Dermal	1			
*Inga* sp.; Banana makako, Caseleira; 043101					5	0.12		
N	Fresh fruits	F			2			
Fu		W	For charcoal		3			
*Millettia* cf. *versicolor* Baker; Pau ferro, Mbota, Mbandu; +; 043220					11	0.27		M, C
Fi		B/BF	E.g. used to tie off leg		1		-	
M	Measles	B		Tie around wrist	2		-	
M	Backache and rheumatic pain	L	Decoction with *D. ambrosioides, O. gratissimum* and *Persea americana*	Steam bath	1		o	
H		W	E.g. to produce mortars		5		+	
Fu		W	For charcoal production		2		+	
*Senna alata* (L.) Roxb.; *; 042691					3	0.07		M, C
M	Skin disease	L	Crushed leaves	Dermal	1		+	
M	Leprosy	L	Balm from roasted leaves and oil	Dermal	1		+	
M	Headache	L	Crushed leaves	Compress around head	1		+	
*Senna occidentalis* (L.) Link; Maniokanioka; *; 044093					6	0.15		M
M	Abdominal pain	R, F	Cold water extract	Oral, suitable for persons aged 6 years and up	4		+	
M	Eye disease (e.g. parasites)	L	Liquid from crushed leaves	Applied to eye	4		+	
*Tephrosia vogelii* Hook.f.; +; 043209					1	0.02		C
Ot	Fish poison	L	Cold water extractfrom crushed leaves		1		o	
Loranthaceae								
*Phragmanthera* sp.; Kinama, Nama; F2399; 043159					2	0.05		
M	Fertility		In combination		2			
MALVACEAE								
*Adansonia digitata* L.; Kibaba; +					3	0.07		M, C
H		W			1		+	
N	Fresh/dried fruit	F	Fresh or dried pulp or juice		1		+	
Fu	Charcoal	W			1		o	
*Cola acuminata* (P.Beauv.) Schott & Endl.; Coleira; +; F06-1664					2	0.05		M, C
N	Fresh/dried fruit	S	Seeds eaten to accompany alcoholic drinks. To alleviate the bitter taste, fruits are sometimes put into termite nests prior to consumption.		2		+	
*Gossypium barbadense* L.; Algodeiro, Husu; -(*); 042775					2	0.05		M, C
M	Abdominal pain	L	Decoction	Oral	1		o	
M	Earache	F	(A) Boil unripe fruit and apply juice into ear (B) Rub unripe fruit in hands and stick into ear		2		+	
*Sida acuta* Burm.f.; Lumzumzu; -(*); 042791					4	0.10		M, C
M	Hepatitis	L	(A) Liquid from crushed leaves (B) Cold water extract from crushed leaves	(A) Applied to eye (B) enema	2		-	
M	Malaria	L	Cold water extract from crushed leaves	Enema	1		o	
M	Joint swelling, Build-up of fluid				1		+	
*Triumfetta cordifolia* A.Rich.; Kingongi, Luvunga (Pl. Mpunga); + (var. tomentosa); 044030					4	0.10		--
Fi		BF			3		+	
M	Pregnancy	L	Decoction	Enema, to “clean” fetus	1		o	
*Triumfetta rhomboidea* Jacq; Ginsunsu branco; +; 042835					1	0.02		M
Fi		BF			1		/	
*Urena lobata* L.; Makolokosso, Gingonge, Ginsunsu; +; 042697					5	0.12		M, C
M	Fertility	L	With Kafuke (Asteraceae indet.)	Enema	2		+	
M	Childhood disease (general weakening)	L		Enema	1		o	
Fi		BF			2		+	
MELASTOMATACEAE								
*Tristemma mauritianum* J.F. Gmel.; +; 042665					2	0.05		--
N	Fresh fruit	F			2		/	
Meliaceae								
*Azadirachta indica* A.Juss.; Neem; -(*)					1	0.02		M, C
M	General	L	Directly consumed or infusion	Oral	1		o	
Moraceae								
*Artocarpus altilis* (Parkinson ex F.A.Zorn) Fosberg; Fruta Pão; -(*); 042674, F19-2542					2	0.05		M, C
N	Cooked fruit	F	Cooked		1		+	
Fo		F, L	For pigs		1		+	
Myrtaceae								
*Psidium guajava* L.; Goiabeira; -(*); 042660					9	0.22		M, C
N	Fresh fruit	F			4		+	
M	Toothache	L	Decoction with *A. cordifolia* and *C. schweinfurthii*	Rinse in mouth	1		+	
M	Diarrhoea	L	Directly consumed	Oral	1		+	
M	Abdominal pain	L	Directly consumed	Oral	4		+	
*Syzygium guineense* (Willd.) DC.; Nkizu; +; 043108					3	0.07		M, C
M	Diarrhoea	R	Cold water extract of crushed leaves, sometimes with *Hymenocardia acida*	Oral	2		+	
M	Fertility	R	Cold water extract of crushed leaves	Oral	1		o	
Ochnaceae								
*Ochna* cf. *afzelii* subsp. *mechowiana* (O.Hoffm.) N.Robson; Coxianti; 043153					2	0.02		M, C
M	Abdominal pain	L	With *X. aethiopica, M. myristica* and Kupidi (*Piper* sp.)		1		-	
M	Backache	L	With *X. aethiopica, M. myristica* and Kupidi (*Piper* sp.)		1		-	
Orobanchaceae								
*Sopubia lanata* Engl.; Diamba dia kana; + (subsp.); 042703					3	0.07		--
M	Gynaecological disorder	L	Suppository from crushed leaves mixed with e.g. garlic	Rectal	1		/	
M	Measles	P	Crushed material	Dermal or enema	2		/	
Oxalidacaeae								
*Oxalis latifolia* Kunth; Banana folha; -(*); 044096					1	0.02		--
M	Anaemia	L	Directly consumed	Oral, also during pregnancy	1		/	
Passifloraceae								
*Passiflora edulis* Sims; Maracujá; *					2	0.05		M, C
N	Fresh fruit	F			2		+	
Phyllanthaceae								
*Bridelia* cf. *ferruginea* Benth.; Nkánkati, Muindu, Windu, Nkalakala; +; 043240					11	0.27		M
M	Worms	B, R	Cold water extract from crushed plant material	Enema	3		o	
M	Abdominal pain	L	Infusion	Oral	1		+	
M	Diarrhoea	B, R	Infusion from crushed plant material	Oral	2		+	
M	Wounds	B, R	(A) Crushed material mixed with small amount of water; (B) Crude bark	Applied to wound	3		o	
Ot	Tobacco	F, L	Dry and mix with tobacco		2		-	
D	Red dye	B			1		+	
*Hymenocardia acida* Tul.; Luvete (Pl: Mpete); +; 043186					7	0.17		M, C
M	Worms	L	With *Bridelia ferruginea*	Enema	1		+	
M	Diarrhoea	R	With *Syzygium guineense*		1		+	
M	Skin disease	B	Balm from powder mixed with oil		2		+	
M	Backache				1		o	
M	Abdominal pain	L, B	Directly consumed	Oral or enema	1		+	
Fu	Firewood	W			1		+	
*Hymenocardia ulmoides* Oliv.; Nkalangangula; +; 043194					4	0.10		--
M	Abdominal pain	L	Directly consumed (young reddish leaves)	Oral	2		o	
M	Diarrhoea	L	Directly consumed (young reddish leaves)	Oral	1		+	
M	Nausea	L	Directly consumed (young reddish leaves)	Oral	1		-	
M	Hunger	L	Directly consumed (young reddish leaves)	Oral	1		-	
H		W			1		+	
Piperaceae								
*Piper* sp.; +; Kupidi					3	0.07		
M	Worms	S	Infusion with e.g. *X. aethiopica, M. myristica* and Ndungu za nzó (*Aframomum* sp.)	Oral	1			
M	Abdominal pain	L	With *X. aethiopica, M. myristica* and *Ochna* cf. *afzelii* subsp. *mechowiana*		1			
M	Backache	L	With *X. aethiopica, M. myristica* and *Ochna* cf. *afzelii* subsp. *mechowiana .*		1			
Poaceae								
*Hyparrhenia* sp.; Capim, Maxinde, Musoki; 043992					6	0.15		
H		L	For roofing, also for walls		4			
Fu	Firelighter				1			
Fo		L	Pasture (burn regulary for young shoots)		1			
*Imperata cylindrica* (L.) Raeusch.; Kindonga; +; 044098					5	0.12		M, C
M	Diabetes	Rh	Decoction	Oral	1		+	
M	Burns	S	Balm (with palm oil)	Dermal	1		o	
H		L	For roofing		4		+	
*Setaria megaphylla* (Steud.) T.Durand & Schinz; Makangaya Madianga; +; 043246					2	0.05		M
Fo		L	For pigs and rabbits		2		+	
Primulaceae								
*Maesa* sp.; Nkambakiana; F07-1771					3	0.07		
M	Childhood disease (blood loss)		With *Euphorbia pulcherrima*		1			
M	Abdominal pain	B			1			
M	Infertility	B	With *Phragmenthera* sp.		1			
T		B			1			
Rubiaceae								
*Gardenia ternifolia* subsp. *jovis-tonantis*(Welw.) Verdc.; Kidia, Kilemba nzau; 043241					10	0.24		M
M	Childhood disease (Gota)	S	Directly consumed, sometimes with *S. anceps*, *V. madiensis*	Oral	2		-	
M	Hernia	R	Cold water extract	Oral	1		-	
M	Measles	F, B, S	(A) Decoction, (B) Crushed seeds	(A, B) Oral or Enema	3		-	
M	Diabetes				1		+	
R	Lightning arrester	P	Planted close to house/ branch placed on roof		2		+	
R	Protection against bad spells	P	Planted in surroundings of house or field		1		-	
R	Nightmares	F	Placed under pillow		1		-	
indet.; Ngolanti, Mamunguamungua; 044021					1	0.02		
M	Potency	B	Decoction	Oral, 2 cups daily	1			
M	Epilepsy	B	Decoction	Oral, 2 cups daily	1			
*Morinda lucida* Benth.; Mazige, Nsiki; +; 043215					4	0.10		M, C
M	Abdominal pain	B, L	Infusion or directly consumed	Oral (very bitter)	3		+	
M	Diarrhoea				1		+	
M	Backache	B, L	Crushed material	Dermal, applied to back	1		+	
*Mussaenda arcuata* Poir.; Nsilu-nsilu; +; 042654					2	0.05		C
M	Hepatitis	L	Crushed leaves	Dermal	1		-	
M	Skin disease	L	Crushed leaves	Dermal	1		+	
*Sarcocephalus latifolius* (Sm.) E.A.Bruce; Kelolo, Kilolwa grande; +; 043154					16	0.39		M, C
M	Abdominal pain	R	(A) Cold water extract or decoction (leave in water for 1 h) (B) directly consumed	(A) Oral or enema, (B) Oral	8		+	
M	Worms	R, B	(A) Directly consumed (B) cold water extract (C) decoction	(A,B) Oral (C) Enema	3		+	
M	Potency	R	(A) Directly consumed (B) Decoction	(A) Oral (B) Oral or enema	1		+	
M	Pains	R	(A) Directly consumed (B) Decoction	(A) Oral (B) Oral or enema	1		+	
M	Typhoid fever	R	Decoction	Oral	1		-	
M	Birth	R	Cold water extract	Oral	1		-	
M	Respiratory disease	B	Directly consumed	Oral	1		+	
Fu	Firewood	W			1		+	
R	Protection	F	Put on baby		1		-	
T		L			1		-	
Rutaceae								
*Zanthoxylum gilletii* (De Wild.) P.G.Waterman; Ndansia tenga; +; F15-2398, F15-2396					1	0.02		C
M	Pregnancy	L	Cooked, young leaves, strengthing	Oral, mixed into meals	1		+	
Smilacaceae								
*Smilax anceps* Willd.; Gipolo, Mpolo, Mukulu; +; 042702					3	0.07		--
M	Cough	L	Infusion	Oral	1		o	
M	Gota	L	With *V. madiensis* and *G. ternifolia*		1		o	
M	Epilepsy	L, R	Decoction	Bath or enema	1		-	
Solanaceae								
*Datura metel* L.; *; 042714					3	0.07		M, C
Ot	Intoxicant	S	Burn	Inhalation	1		+	
M	Rheumatism	S	With other species		1		-	
M	Inflammation	L	Crushed leaves	Dermal	1		+	
*Solanum aculeastrum* Dunal; Mabumi, Gituno; + (var.); 043132					2	0.05		M, C
M	Childhood disease (Baço)	F, B	Decoction	Enema or oral	2		-	
*Solanum americanum* Mill.; Lundumbo, Ndumbo; *; 043164					3	0.07		M
N	Cooked leaves	L	Cooked and eaten as a vegetable		2		-	
M	Abdominal pain	L	Cold water extract of crushed leaves	Oral	1		o	
*Solanum mauritianum* Scop.; Daniele; *; 044100					1	0.02		M
M	Cough	L	Decoction	Steam bath or inhalation	1		-	
*Solanum nigrum* L.; Gizue, Lundumbo, Windangonge; *; 043139					3	0.07		M, C
M	for Babys	L	Sap from crushed leaves	Few drops daily applied to umbilical cord	1		o	
M	Fever	L	Infusion	Oral, suitable for children	1		+	
M	Diarrhoea	R			1		+	
Urticaceae								
*Laportea* sp.; Kihidi, Hidi; 043157					3	0.07		
M	Cough	L	Infusion	Oral suitable for children	2			
M	Haemorrhoids	L	Cooked	Oral, eaten with peanuts	1			
Verbenaceae								
*Lippia multiflora* Moldenke; Bulukutu; +; 044101					2	0.05		M, C
T		L	Infusion		2		+	
*Stachytarpheta cayennensis* (Rich.) Vahl; Kalangue; -(*); 043188					3	0.07		M, C
M	Malaria		Crushed material	Rectal, suppository	1		-	
M	Diarrhoea		Decoction	Oral	1		-	
M	Joint swelling, Build-up of fluid	L	Infusion	Oral	1		-	
M	Skin disease	L	Infusion	Oral	1		o	
M	Respiratory disease	L	Infusion	Oral	1		-	
Vitaceae								
*Cayratia gracilis* (Guill. & Perr.) Suess.; Nlembuzi; +; 043147					1	0.02		--
M	Joint swelling, Build-up of fluid	L	Crushed leaves	Dermal, applied to swollen joints	1		o	
*Cayratia* sp.; Hoselia, Uuse; 043166					2	0.05		
M	Anaemia	L	Cooked	Oral	1			
N	Cooked leaves	L	Cooked and eaten as a vegetable		1			
*Cissus rubiginosa* (Welw. ex Baker) Planch.; Nkokelakai, Mukokelakai; +; 043207					4	0.10		M
M	Fever	L	Crushed leaves added to bathwater	Bath,suitable for children	1		-	
M	Childhood disease (Baço)	L	Crushed leaves	Enema	2		-	
M	Worms	L	Crushed leaves	Enema	1		-	
M	Rheumatism, leg pain	L	Decoction	Enema	1		+	
indet.; Dizo dia lunguenia; 043122					1	0.02		
M	Poisoning	St	-	Used to tie off poisoned limb	1			
Xanthorrhoeaceae								
*Aloe buettneri* A.Berger; Badianseka; +; F05-1633					7	0.17		M
M	Skin disease	L	Sap from cut leaf	Dermal	3		+	
M	Potency	L	Decoction	Oral or Enema	1		-	
M	Fertility	L	Decoction	Enema	1		o	
M	Worms	L	Decoction	Enema	1		+	
M	Backache	L	Decoction	Oral or Enema	1		-	
M	Respiratory disease	L	Directly consumed or decoction	Oral, contains many vitamins	1		+	
M	Blood-purifying	F	Directly consumed	Oral	1		-	
N	Cooked leaves	L	Eaten with beans as a vegetable		1		-	
Zingiberaceae								
*Aframomum alboviolaceum* (Ridl.) K.Schum.; Ginguenga da queimada, Masunjá. Mansassa, Dimbomboa; +; F19-2561, F01-1105					14	0.34		M
N	Fresh fruit	F	Directly consumed or as juice		9		+	
M	Yellow fever	Rh	Crushed rhizome	Enema	1		-	
M	Headache	L	Sap from chewed material	Applied to eyes	1		+	
M	Hepatitis	Rh			1		-	
M	Respiratory disease	Rh	With root of *A. senegalensis*		1		+	
M	Epilepsy	Rh L	Decoction	Bath or Enema	1		-	
*Aframomum* sp.; Ndungu za nzó; F18-2534					2	0.05		
M	Worms	S	Infusion with i.e. Kupidi (*Piper* sp.) *M. myristica* and *X. aethiopica*	Oral	2			
*Zingiber officinale* Roscoe; Gengibre, Gibidi; -(*)					3	0.07		M, C
M	Respiratory disease	Rh			2		+	
M	Fever	Rh			1		+	
Ot		Rh	As condiment in palm wine “Maruwu”		1		o	

Legend: All species identified until family level are listed with scientific and local name and details to representation in Angolan checklist [31]: “+” listed, * listed as naturalised; (*) not listed in checklist, but known to be naturalised in Angola. If herbarium specimens were entered into Herbarium Dresdense, the codes are listed in the table. If it wasn’t possible to prepare specimens or if the material was damaged during transportation, numbers of the foto documention are mentioned, marked by ‘F’. ^a^ *UC* Use categories, *D* Dye, *Fi* Fibres, *Fo* Forage, *Fu* Fuel, *H* Handicraft/building material, *M* Medicine, *N* Nutrition/food, *O* Ornamental, *R* Ritual/Magic, *T* Tea, *Ot* Other; ^b^Used plant parts: *B* Bark, *BF* Bast fibres, *F* Fruit, *L* Leaves, *Ms* Milksap, *P* Whole plant, *R* Root, *Re* Resin, *Rh* Rhizome, *S* Seed, *St* Stem, *W* Wood; ^c^ *NUR* Number of use-reports per plant and citations per usage; ^d^ *CI* Cultural Importance Index; ^e^ *L* Comparision to [44] for medical uses or to [4–6] for other than medical uses: “ + ” use listed; “o” similar use listed; “-” use not listed; “/” species not listed; ^f^ *DB* Result of PubMed-Database-Inquiry in June 2015: (M) medical studies performed, (C) chemical analysis performed

On average, 4.1 different use-reports per species were documented, 72.1 % of which refer to medical treatments. 10.6 % of uses refer to food plants. Other use categories covered are handicraft and building material (3.6 %), tea (non medical, 2.6 %), fuels (1.8 %), forage plants (1.8 %), ritual uses (1.8 %), source of fibers (1.4 %), ornamentals (1.0 %), dyes (1.0 %), and others (2.2 %).

Of the documented plants, 41.3 % were shrubs or trees, 9.1 % subshrubs, 24.8 % perennial herbaceous plants, 13.2 % annuals and 11.6 % annual or perennial climbers. Due to regular fires many species that are documented in the literature as trees, showed a shrubby habit in the study area. The most commonly used plant parts are leaves or fronds (224 citations for 78 species), fruits (73 citations for 35 species, including seeds) and underground organs, such as roots, root tubers and rhizomes (75 citations for 28 species).

NUR and CI were calculated for each species to evaluate the importance of a particular plant species and to standardize the data. NUR varies between 1 and 16, with an average value of 3.63. The related value of CI covers a range from 0.02 to 0.39 with an average of 0.09. Table 2 summarises the ten highest-ranking species. The highest values were reached by *Annona senegalensis* Pers. and *Sarcocephalus latifolius* (Sm.) E.A. Bruce with NUR = 16 and CI = 0.39.

**Table 2:** Overview of the ten species with highest number of use-reports (NUR) and cultural importance index (CI)

Species	NUR	UC	SUC	PP	CI
*Annona senegalensis*	16	3	10	4	0,39
*Sarcocephalus latifolius*	16	4	10	5	0,39
*Aframomum alboviolaceum*	14	2	6	3	0,34
*Ocimum gratissimum*	11	1	6	1	0,27
*Millettia* cf. *versicolor*	11	4	5	4	0,27
*Bridelia* cf. *ferruginea*	11	3	6	4	0,27
*Dysphania ambrosioides*	10	1	9	1	0,24
*Vitex madiensis* subsp. *madiensis*	10	4	6	2	0,24
*Erythrina abyssinica*	10	1	5	1	0,24
*Gardenia ternifolia* subsp. *jovis-tonantis*	10	2	7	5	0,24

Legend: *UC* Number of use categories, *SUC* Number of sub-categories, *PP* different plants parts used

For all specimens determined to species level, literature comparison was carried out to emphasize hitherto unknown use-reports. 7.6 % of citations referred to species not documented in the ethnobotanical literature used for comparison and 26.6 % of the reports added new uses to species that were already known for their ethnobotanical relevance.

### Medicinal plants

**Fig. 3:**
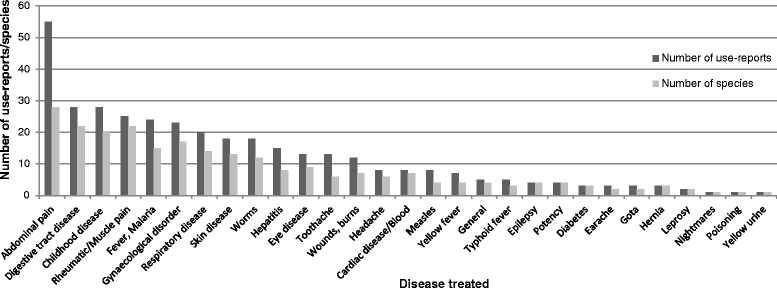
Analysis of medical use-reports ordered by disease or disease pattern. Number of species and use-reports for different disorders

The high percentage of medical use-reports allowed further analyses of the diseases treated with traditional medicine. As can be seen in Fig. 3, medical plants are used against both specific diseases, such as typhoid fever or diabetes, and widespread symptoms like abdominal pain. For reasons of clarity, some symptoms listed separately in Table 1, are merged in Fig. 3 by means of the body systems affected. E. g., “digestive tract diseases” includes constipation, diarrhoea and nausea; “gynaecologic disorder” merges menstrual disorders with fertility and pregnancy disorders. As abdominal pain is a widespread symptom which might be caused by several diseases, this use category was kept separately from related categories, such as digestive tract or gynaecological disorder, hepatitis or urinary tract infections, if no clear attribution was made by informants. The most frequently listed areas of application are abdominal pain (55 use-reports), digestive tract diseases (28 UR), childhood diseases (28 UR), rheumatic or muscle pain (25 UR) and fevers (25 UR). Two diseases mentioned in interviews could not be identified and were therefore not translated. *Baço* refers to a disease affecting mostly children, including symptoms that might be related to splenomegaly after malaria infection. *Gota* would be literally translated as gout, but is commonly described as a childhood disease with epileptic symptoms.

The most frequently cited preparation methods for medical plants are decoctions (72 citations) and freshly crushed material, as it is used for dermal administration or to extract the sap from a tissue (71 citations). Furthermore, use-reports include infusions (59 citations), direct consumption of the fresh plant material (50 citations), macerations (27 citations) and other methods, such as burning or drying of the material (4 citations).

Treatments are mostly accomplished through the enteral route by oral intake (139 citations). Rectal drug administration is also common (68 citations), especially by enema. Other methods documented include inhalation (21 citations), ophthalmic drugs (13 citations) and the uptake through oral mucous, e. g. by rinsing (12 citations). Nasal (2 citations) and otic (1 citation) treatments were less frequently cited.

**Fig. 4:**
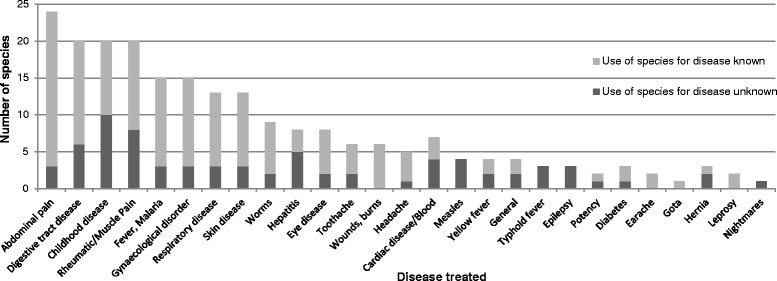
Analysis of medical use-reports ordered by disease or disease pattern. Number of identified species used for different disorders and percentage of new use-reports (dark fraction). Only specimens identified until species level were included in the figure. This explains aberrations to Fig. 3

As stated before, literature comparison was carried out for all species determined to species level, to emphasize hitherto unknown use-reports. Figure 4 shows the percentage of species whose use for a specific disease pattern was new to the studied literature. The percentage of new species was especially high in categories linked to a specific disease, such as measles, typhoid fever and epilepsy; whereas it was lower for unspecific symptoms i.e. abdominal pain.

### Food plants

During our study, we recorded 53 use-reports for 23 species that are used as food plants. The most commonly cited species are *Aframomum alboviolaceum* (9 citations), *Landolphia owariensis* (6 citations), *Crassocephalum rubens* (5 citations), *Pteridium centrali-africanum* (4 citations), and *Psidium guajava* (4 citations).

Fruits are the most frequently used plant parts for nutrition; they are mostly eaten raw (32 citations) or less often cooked or heated in hot water before consumption (6 citations). 13 use-reports mention cooked leaves that are used as a vegetable. Cooked root tubers or raw stems are both documented in only one use-report.

Although the documented species might be found naturalized within the disturbed savannahs of the region, in some cases we also observed cultivation, e.g. for *Dacryodes edulis, Canarium schweinfurthii, Cajanus cajan, Artocarpus altilis, Psidium guajava* and *Passiflora edulis.*

Not all plants were incorporated into diets under normal conditions. For example, the taste of *Pteridium centrali-africanum* fronds was described as rather unpleasant by some of the respondents. For *Crassocephalum rubens* informants mentioned that they know the leaves are edible and can be used as a vegetable, but do not use them as long as other leafy vegetables such as *Manihot esculenta* or *Amaranthus* spp. leaves are available. Similar explanations were documented for the root tubers of *Dioscorea dumetorum.* Food plants such as these, which are mainly consumed during times of food shortage are often referred to as famine foods [46].

It has already been shown in other studies for sub-Saharan Africa that some fruits are not harvested in great amounts but rather consumed as a ‘snack’ while spending time in the field [9, 47]. The fruits of *Annona senegalensis, Anisophyllea quangensis, Vitex madiensis* subsp. *madiensis, Tristemma mauritianum* and the stem of *Costus afer* fit to this description.

In contrast, the fruits of *Landolphia owariensis, Canarium schweinfurthii, Dacryodes edulis, Adansonia digitata, Psidium guajava, Passiflora edulis* and *Aframomum alboviolaceum* were sometimes harvested in greater quantities and sold at local markets. Except for *Landolphia owariensis, Adansonia digitata* and *Aframomum alboviolaceum*, these plants are also in cultivation, but may have been abandoned or escaped, and are now growing in the surroundings of the villages and fields.

### Other uses

As stated above, other use categories were mentioned less frequently in comparison to medicinal and food plants (in total 55 citations, 17.2 %). Still, the citations in these categories cover a wide field of different applications, including e. g. fibres and dyes, fuel, building material, ritual and ornamental uses. About 60 % of citations in these use-categories have already been documented in literature about local plant use. Percentage of already known citations is especially high (100 %) in the categories dyes (2 species), fuels (4 species) and handicraft/building material (6 species), which include many woody species. The highest amount of hitherto unknown plant uses was documented in the use-category ‘Ornamental’, where four of five species haven’t been found in the literature used for comparison. Presumably, this is due to the fact that many ethnobotanical studies do not include ornamental uses.

## Discussion

Although savannahs are not the potentially natural vegetation in the study area, they are of high importance to Bakongo people. The 122 documented species are used for diverse applications, ranging from medical treatments and handicrafts to ritual uses. The anthropogenic disturbance of the potential natural vegetation in the area has already been documented in 1970 by Barbosa [19], the incorporation of the plants into livelihood strategies is therefore representing the expectations.

We documented a high amount of use-reports that were new to the literature used for comparison (34.0 % of citations). Still, the documentation of useful plants in Uíge cannot be considered completed. As time for fieldwork was limited, some plants couldn’t be identified until family level and were therefore not included in the documentation. Also, to obtain reliable data, we spatially restricted the study area in order to include a variety of informants with different backgrounds, age and gender from every village. The high percentage of unknown plant uses emphasizes the potential of the study area for further ethnobotanical findings and can be considered as a basis for future research in the region.

### Medicinal plants

The high amount of medical use-reports (71 %) indicates that plants still play a crucial role in rural health care. This impression is strengthened by the diversity of illnesses treated with plant preparations. As already mentioned before, this is probably due to the difficult access to medical care.

The results regarding preparation and administration of plant remedies are in line with other ethnobotanical works. Decoctions, raw plant parts and infusions as the most common preparation methods and oral administration as the most frequent administration route are documented in many studies for sub-Saharan Africa [47–52].

As mentioned before, 34.0 % of all recorded use-reports were not documented in the literature used for comparison; in the use-category of medicinal plants the percentage of previously unknown use-reports is comparable (33.8 %). To identify promising targets for further biochemical investigation, we additionally conducted a search in the Pubmed-database [53], as documented in Table 1. For the majority of the species (58.4 %) both medical and phytochemical studies have already been carried out. Further 24.8 % have been analysed either through medical or phytochemical studies. For 17 species (16.8 %) the inquiry did not reveal further information. As 14 of these species are used in traditional medicine in Northern Angola, they might be promising targets for further studies (Table 3).

**Table 3:** Species with medical uses not yet tested pharmacologically. Name and overall cultural importance (CI) of species are given

Species	CI
*Alvesia rosmarinifolia*	0.02
*Anisophyllea* cf. *quangensis*	0.05
*Cayratia gracilis*	0.02
*Desmodium velutinum*	0.02
*Gymnanthemum cf. glaberrimum*	0,10
*Hymenocardia ulmoides*	0.10
*Lannea* cf. *antiscorbutica*	0.10
*Oxalis latifolia*	0.02
*Pleiotaxis rugosa*	0,07
*Pteridium centrali-africanum*	0.12
*Smilax anceps*	0.07
*Sopubia lanata*	0.07
*Triumfetta cordifolia*	0.10
*Vernonella* cf. *subaphylla*	0.12

Most of the species with high CI-values have already been evaluated in pharmaceutical or medical surveys. Few studies have been conducted for *V. madiensis* subsp*. madiensis. G. ternifolia* subsp*. jovis-tonantis* and *A. alboviolaceum*. Especially *G. ternifolia* subsp*. jovis-tonantis* and *A. alboviolaceum* might be an interesting object of further investigation, as they are used to treat severe illnesses such as measles, diabetes, yellow fever, hepatitis or epilepsy.

### Food plants

The documentation of food plants does mainly meet the expectations. The prevalence of fresh fruits in this use-category as well as the phenomena of famine foods and snack plants have already been documented in other studies [9, 46]. Our findings support the widely-represented concept that food plants collected in the wild are well incorporated into local livelihood strategies in rural, tropical Africa and may contribute to food-security and socio-economic sustainability [46, 54].

Most of the plants reported in this use-category have already been documented as food plants. In the literature used for comparison, only *Solanum americanum* leaves, *Tristemma mauritianum* fruits, *Costus afer* stems, *Aloe buettneri* leaves and *Jatropha curcas* fruits have not been listed yet. In the cases of *Tristemma mauritianum* and *Solanum americanum* the use as a food plant has been documented in other African regions [54–56]. In contrast, *Costus afer* is well known as a medicinal plant [44, 57], but we found no evidence for a use as ‘snack food’ in the literature. Chemical analysis of the stem shows that there are no toxic effects to be expected and that the plant is a good source of magnesium and potassium [58]. Similarly, *Aloe buettneri* is a well-known medicinal plant in Africa, whereas documentation for use as a food plant is scarce; only one publication mentions the use of the flowers in soups [59]. Still, it is known that the leaves of other *Aloe* species may be cooked as a leafy salad, probably after the green leaf tissue has been peeled off to relieve the bitter taste [59]. In contrast, the use of *Jatropha curcas* fruits as a food is questionable, as they are known to be toxic [60]. There exist publications about non-toxic accessions of the plant, but as they are mainly documented for Mexico [61, 62], it is unlikely that these accessions are grown in the study area. As this use was only mentioned by one informant, we cannot rule out the possibility of inaccurate information. As already stated in the result section, not all citations referred to plants that are regularly used, so we could make no observations about the consumption of *Jatropha curcas* fruits.

### Other uses

In comparison to medicinal and food plants, other use categories were mentioned less frequently. The small proportion of use-reports for handicraft and building material or fuels might be attributed to the lack of large trees in degraded landscapes. Other uses often seem to be replaced by industrial products, which might lead to a loss of traditional knowledge. As could be observed in the surroundings of Uíge city, traditional thatching materials are often replaced by corrugated sheet metal. Similarly, the knowledge about natural dyes was still present, although informants stated that they hadn’t used them by themselves.

### Impact of disturbance and implications for conservation

The species of highest cultural importance, as listed in Table 2, include no species of special concern. All plants listed are readily available in the region: *A. senegalensis*, *S. latifolius*, *A. alboviolaceum, B. ferruginea, V. madiensis* subsp*. madiensis, E. abyssinica* and *G. ternifolia* subsp*. jovis-tonantis* belong to the typical vegetation of disturbed savannahs, while *O. gratissimum*, *M. versicolor* and *D. ambrosioides* are rather found in the transition zones to village outskirts.

It is conspicuous that seven of the ten highest ranked plants are woody species. These values might be attributed to the fact that trees and shrubs offer a greater variety of useable parts than herbaceous plants, e. g. wood and bark [41, 63, 64] and that woody plants are available throughout the year [9, 65]. In the context of disturbance vegetation, other reasons might be the greater visibility of trees and shrubs in grasslands as well a better resistance of mature trees to fire in comparison to many herbaceous plants [9, 13].

Nevertheless, the amount of annual and perennial herbaceous plants is still high (49.6 %). This number might be surprising, considering that the *Hyparrhenium* species, which dominate the savannahs of the region, form compact eyries and reach heights up to 2.5 m and therefore inhibit the growth of small herbaceous plants. From that point of view, anthropological disturbance actually might contribute to a higher biodiversity in anthropogenic savannahs. Grazing, agriculture and small savannah fires can create a mosaic of small-scale habitats with different light conditions and therefore permits the growth of herbaceous, short-lived species and pyrophytic plants [66, 67]. It has been shown that some traditional fire management practices are specifically aimed at the creation of such small-scale habitats (patch-mosaic burning) [68].

Although no such traditional fire management systems are known for the north of Angola, we observed that some of the used species, e. g. *Helichrysum mechowianum, Pleiotaxis rugosa, Baccharoides guineensis* or *Smilax anceps*, often grew in recently burnt areas, where light exposure was high. Likewise, the young fronds of *Pteridium centrali-africanum* were regularly found in recently burnt areas, where proliferation is promoted. Similarly, fruits of *Aframomum alboviolaceum* were often collected in recently burnt savannahs. This might be related to the fact that a thick grass cover does not only inhibit growth of small herbaceous plants, but also impedes access for plant collection. Two informants explained during semi-structured interviews that sometimes grassland is specifically burnt to facilitate access to *Aframomum alboviolaceum* fruits, which may be sold at local markets. Therefore, even if the above-mentioned species are not critically endangered, their collection may exert high pressure on the ecosystem, especially when large areas are exposed or fires are repeated within short time periods [69]. Besides, for some woody species, e. g. *E. abyssinica, B. ferruginea* and *S. latifolius,* fire resistance was observed to be severely affected by intensive collection of roots and bark. Loss of bark may lead to higher vulnerability towards fungal infection and pests, and decrease fire and drought resistance and by that means lead to further reduction of woody species in the area [70, 71].

As can be noticed by those examples, the analysis of plant use and cultural importance of plants may help to understand land use and fire management practices and to develop conservation strategies to ensure the access to useful plants for future generations. However, a complete analysis of that topic must take many other aspects into account, e.g. economic, ritual or agricultural issues.

## Conclusion

Despite the comparably low biodiversity, plants from degraded savannah vegetation in Uíge, Angola, are incorporated in manifold livelihood strategies of the local population. Savannahs are sources of food and medicine; they provide plants for handicraft, forage and fuel as well as ritual and ornamental plants. While some uses, e. g. dyes, fibres and building material often seem to be replaced by industrial products, we recorded a high amount of medicinal plants (70 %). As access to health care especially in rural areas is difficult, plants still play a vital role in this field.

As a result of the diverse incorporation of plants in livelihood strategies the north of Angola turns into a promising region for further ethnobotanical research. This is underlined by the high amount (34.0 %) of use-reports that have not been documented in the literature used for comparison.

Especially in the field of medicinal plants, further investigation is necessary to evaluate the pharmacological potential of the plants and to improve self-medication practises in the future.

Our study highlights 14 species which are especially interesting for pharmacological analysis. It is essential to communicate the results of ethnobotanical and pharmacological research to the rural population. In the framework of the project, the establishment of a botanical garden based on ethnobotanical criteria is envisaged. The presented study encourages those efforts, as it both points out the richness of ethnobotanical knowledge in the region and the importance of plant remedies in rural health care.

The species of highest cultural importance do not include plants of special concern. Still, collection of plants can exert high pressure on the ecosystem, as sometimes fires might be set to improve accessibility of savannahs. In order to conserve the savannahs as a source of useful plants, further studies should take a closer look at plant collection and traditional fire management systems.

## Abbreviations

CI: Cultural importance index
NUR: Number of use-reports
UR: Use-report
